# Evidence for recombination between a sialidase (*nanH*) of *Actinomyces naeslundii* and *Actinomyces oris*, previously named ‘*Actinomyces naeslundii* genospecies 1 and 2’

**DOI:** 10.1111/j.1574-6968.2008.01336.x

**Published:** 2008-09-24

**Authors:** Thuy Do, Uta Henssge, Steven C Gilbert, Douglas Clark, David Beighton

**Affiliations:** King's College, School of Medicine and DentistryLondon, UK

**Keywords:** *Actinomyces*, sialidase, dental plaque

## Abstract

*Actinomyces* spp., predominant members of human oral biofilms, may use extracellular sialidase to promote adhesion, deglycosylate immunoglobulins and liberation of nutrients. Partial *nanH* gene sequences (1077 bp) from *Actinomyces oris* (*n* =74), *Actinomyces naeslundii* (*n* =30), *Actinomyces viscosus* (*n* =1) and *Actinomyces johnsonii* (*n* =2) which included the active-site region and the bacterial neuraminidase repeats (BNRs) were compared. The sequences were aligned and each species formed a distinct cluster with five isolates having intermediate positions. These five isolates (two *A. oris* and three *A. naeslundii*) exhibited interspecies recombination. The nonsynonymous/synonymous ratio was <1 for both *A. oris* and *A. naeslundii* indicating that *nanH* in both species is under stabilizing selective pressure; nonsynonymous mutations are not selected. However, for *A. oris* significant negative values in tests for neutral selection suggested the rate of mutation in *A. oris* was greater than in *A. naeslundii* but with selection against nonsynonymous mutations. This was supported by the observation that the frequency of polymorphic sites in *A. oris*, which were monomorphic in *A. naeslundii* was significantly greater than the frequency of polymorphic sites in *A. naeslundii* which were monomorphic in *A. oris* (χ^2^=7.011; *P* =0.00081). The higher proportions of *A. oris* in the oral biofilm might be explained by the higher mutation rate facilitating an increased ability to respond successfully to environmental stress.

## Introduction

Sialidase activity is produced by many commensal oral bacteria including *Actinomyces* spp. (Moncla & Braham, 1989; Beighton & Whiley, 1990; Beighton *et al.*, 1991; Braham & Moncla, 1992). The activity produced by *Actinomyces* spp. desialylates IgA by the removal of the terminal sialic acid residues rendering the molecule more susceptible to proteolysis (Reinholdt *et al.*, 1990; Frandsen, 1994). Sialidase activity also appears important in the nutrition of the oral biofilm because sialidase activity increases in the absence of host diet, and withdrawal of the normal diet increases the proportions of sialidase-producing bacteria (Smith & Beighton, 1986; Lucas *et al.*, 1997; Sheehy *et al.*, 2000). Sialic acid is utilized by various oral streptococci, *Actinomyces naeslundii* and by different mixed populations of subgingival plaque bacteria (ter Steeg *et al.*, 1987; Frandsen, 1994; Byers *et al.*, 1996, 1999; Homer *et al.*, 1996). Sialidase also exposes galactose residues of O- and N-linked glycans which mediate the adherence of human *A. naeslundii* strains described previously as genospecies 1 and 2 to human glycans including those of the salivary pellicle (Costello *et al.*, 1979; Gibbons *et al.*, 1990).

The complete genome of ‘*A. naeslundii*’ MG1 (http://cmr.jcvi.org/tigr-scripts/CMR/CmrHomePage.cgi/) contains ORFs for two sialidase genes (ANA2709, *nanH*, a sialidase and ANA1493 an exosialidase). The sequence of the *nanH* gene (ANA2709) of MG1 has >97% similarity with the sequences of the two previously reported *Actinomyces* sialidase gene sequences (Henningsen *et al.*, 1991; Yeung, 1993) while the ANA1493 exhibits <5% sequence similarity with the *nanH* genes.

Two of the strains from which the *nanH* was sequenced were described as *Actinomyces viscosus* but such human strains were subsequently described as *A. naeslundii* genospecies 2 (Johnson *et al.*, 1990). However, on the basis of a phylogenetic analysis of partial gene sequences of housekeeping genes, we (Henssge *et al.*, in press) have reported that strains previously identified as *A. naeslundii* genospecies 2, including the sequenced strain MG1, should be reclassified as *Actinomyces oris*. The species *A. naeslundii* has been amended to include only strains previously identified as *A. naeslundii* genospecies 1. Therefore, we have *nanH* sequence data on three *A. oris* strains but no information of the *nanH* genes of *A. naeslundii, Actinomyces johnsonii* (previously *A. naeslundii* genospecies WVA 963) or animal strains of *A. viscosus*.

In this report, we have compared the nucleotide and amino acid sequences of the region of the *nanH* gene containing the active site (Crennell *et al.*, 1993) and the five Asp boxes or bacterial neuraminidase repeats (BNRs, Henningsen *et al.*, 1991) of these *Actinomyces* species. In the mouth *A. naeslundii* and *A. oris* occupy the same sites but *A. oris* is the predominant species (Bowden *et al.*, 1999) while *A. johnsonii* was isolated from the gingival crevice (Johnson *et al.*, 1990). We present a phylogenetic analysis of a partial sequence of *nanH* in these four species and present evidence of interspecies recombination between *nanH* genes.

## Materials and methods

### Bacterial strains

The isolates have all been reported and identified in a previous taxonomic study (Henssge *et al.*, in press) describing the new species *A. oris* (previously *A. naeslundii* genospecies 2) and *A. johnsonii* (previously *A. naeslundii* genospecies WVA 963) and reporting an emended description of *A. naeslundii* (previously *A. naeslundii* genospecies 1). The strains included in this study were 30 *A. naeslundii* (CCUG 33521, CCUG 33522, CCUG 33519, CCUG 33523, ATCC 12104, CCUG 34725, CCUG 35334 and CCUG 37599 and 22 human clinical and oral isolates); 71 *A. oris* (P2G, P5K, P6K, P7K, P8K, P9K, Pn4D, Pn5D, CCUG 33915, CCUG 33919, CCUG 33920, CCUG 33914, CCUG 34285, CCUG 34286 and ATCC 27044 and 56 human clinical and oral isolates); two *A. johnsonii* (CCUG 33932 and CCUG 34287) isolates and one *A. viscosus* (NCTC 10951). The oral and clinical isolates were given study numbers from 1 to 94. All isolates were cultured anaerobically at 37 °C on Fastidious Anaerobe Agar (LabM) supplemented with 5% (v/v) defibrinated horse blood and stored at −80 °C in brain–heart infusion (Oxoid) containing 50% glycerol.

### PCR conditions and *nanH* sequencing

DNA was extracted from cells as described previously (Henssge *et al.*, in press). Owing to the length of the gene fragment which was likely to contain the sites of interest, two pairs of primers were used to obtain a 1077-bp fragment of the *nanH* gene in all species: Sial-F1 5′-ACACGATCACGCAAGCCGA-3′ and Sial-R1 5′-CGACCTTGTTCTCATCCA-3′ and Sial-F2 5′-AACCACATCGTCCA-3′ and Sial-R2 5′-GAGCCAGTTCATCGTGAA-3′. The PCRs were performed using Reddymix (Abgene, Epsom, Surrey, UK) and the PCR conditions for each pair of primers were an initial denaturation for 10 min at 94 °C followed by 30 cycles of 94 °C for 30 s, 49 °C for 30 s and 72 °C for 90 s. A final extension was carried out for 5 min at 72 °C. The PCR products were visualized on a 1% agarose gel stained with Gel Red (Biotium Inc.). The amplicons were cleaned using a 50:50 mixture of 40% polyethylene glycol and 3 M NaCl, washed twice with 70% ethanol and rehydrated in sterile water. The same primers were used for the sequencing reactions and all amplicons were sequenced in both directions using the BigDye Terminator Sequencing kit (Applied Biosystems) and reaction products were run on a 3730xl sequencer (Applied Biosystems).

### *nanH* sequence analysis

The DNA sequences were aligned using bioedit (Wide-field images were captured using an Olympus BX51 upright wide-field microscope with a ×40/1.00 UPlan Apo objective and a Coolsnap ES camera (Photometrics) through MetaVuesoftware (Molecular Devices). All wide-field images were captured using the same exposure and image scaling settings, and image scaling was adjusted to exclude background immunostaining. Offline image analysis used ImageJsoftware (http://www.mbio.ncsu.edu/BioEdit/bioedit.html/). The phylogenetic relationships between partial *nanH* nucleotide sequences of the reference strains, the human oral and clinical isolates and the two ‘*A. viscosus*’*nanH* sequences in GenBank (L06898 and X62276) and the *nanH* of the sequenced strain MG1 (ANA2709) were analysed using mega 4 (Tamura *et al.*, 2007). Distances were calculated using the Kimura two-parameter model and for clustering the neighbour-joining method of Saitou & Nei (1987) using bootstrap values based on 500 replicates was used. The amino acid sequences were clustered using clustalw2 (http://www.ebi.ac.uk/).

dnasp (Rozas *et al.*, 2003) was used to investigate the *nanH* sequences of *A. naeslundii* and *A. oris*. The G+C content of the partial genes sequences, the number of discrete sequences, the number of polymorphic sites, average nonsynonymous/synonymous ratios (d*N*/d*S*) were calculated and nucleotide diversity was estimated by determining π (nucleotide diversity) and θ (the total number of mutations). To test for neutral molecular evolution three tests were used; Tajima's *D*, based on the differences between the number of segregating sites and the average number of nucleotide differences, Fu and Li's D^*^, based on the differences between the number of singletons (mutations appearing only once among the sequences) and the total number of mutations and Fu and Li's F^*^, based on the differences between the number of singletons, and the average number of nucleotide differences between pairs of sequences. The extent of the DNA sequence polymorphism between *A. naeslundii* and *A. oris* was calculated and compared using a χ^2^ test.

Split decomposition trees were constructed with 1000 bootstrap replicates based on parsimony splits as implemented in the splitstree 4.0 (Hudson & Bryant, 2006) and the statistic phi was calculated. To identify strains with evidence of recombination the Recombination Detection Package (Martin *et al.*, 2005) was used with the default settings.

### Identification of BNR sequences and active site residues

The BNRs were identified using the InterProScan Sequence Search tool (http://www.ebi.ac.uk/Tools/InterProScan/). The amino acid polymorphisms in the BNRs were determined by visual inspection. The partial *nanH* amino acid sequences of the four type strains were aligned with the sequence of a *Salmonella typhimurium* sialidase (M55342) using Parallel Protein Information Analysis System (http://www.cbrc.jp/papia/papia.html/) and the 12 putative active site residues of the *S. typhimurium* sialidase (Crennell *et al.*, 1993) were identified.

### Detection of *nanH* transcripts

Each type strain was grown on FAA supplemented with 5% (v/v) defibrinated horse blood and total RNA was extracted using the UltraClean Microbial RNA Isolation Kit (MO BIO Laboratories Inc.). Reverse transcription was performed using the Omniscript RT kit (Qiagen Ltd) with primers, Sial-F1 and Sial-R1, for *nanH* and AtpA-F (CCCTGGAGTACACCACCAT) and AtpA-R (CGCCAGGGTGATCTTGAG), to amplify the housekeeping gene, *atpA* (ATP synthase F1, α subunit, ANA_0169). The thermal cycling was as follows: cDNA synthesis at 37 °C for 30 min, 94 °C for 1 min, followed by amplification with 40 cycles of denaturation at 94 °C for 20 s, annealing at 55 °C for 30 s, extension at 72 °C for 1 min, and then the final extension at 72 °C for 5 min. The products were run on a 1% agarose gel, with Gel Red incorporated, for 10 min at 100 V. The identity of the amplicons was confirmed by sequencing as described above.

## Results

**Fig. 1:**
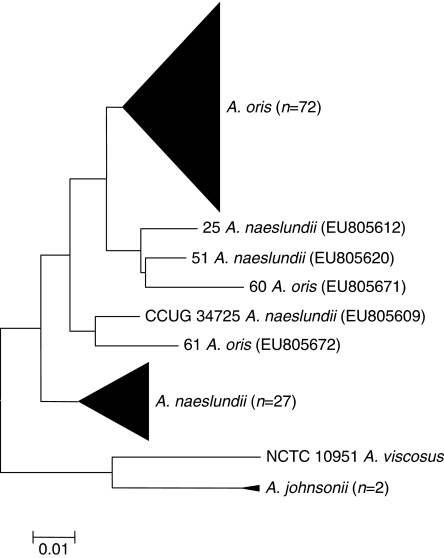
Neighbour-joining tree showing relationships between type and reference strains of *Actinomyces naeslundii, Actinomyces oris, Actinomyces johnsonii* and *Actinomyces viscosus* and oral and clinical isolates determined by partial *nanH* gene sequence analysis. *Actinomyces naeslundii* strains 25, 51 and CCUG 34725 and *A. oris* strains 60 and 61 (each shown with the *nanH* sequence accession number) did not cluster with the majority of strains of the same species and were identified as strains with significant evidence of interspecies recombination. Scale bar=0.01 substitutions per site.

The construction of a phylogenetic tree using the *nanH* sequences indicated that the four species were separated from each other except that two of *A. oris* strains (strains 60 and 61) were separate from the major *A. oris* cluster and three *A. naeslundii* strains (strains 25, 51 and CCUG 34725) were distinct from the major *A. naeslundii* cluster (Fig. 1). Essentially, the same tree topography was found for the analysis of the derived amino acid sequences (data not shown) with the same isolates being found on the periphery of the clusters composed of the majority of the *A. oris* and *A. naeslundii* isolates and clearly distinct from the *A. johnsonii* and *A. viscosus* sequences.

The dN/dS values for *A. oris* and *A. naeslundii* were 0.1466 and 0.2134, respectively. Tajima's *D*, Fu and Li's D^*^ and F^*^ were all significantly negative for *A. oris* but did not achieve significance for *A. naeslundii* (Table 1). The test for DNA divergence between populations implemented in dnasp indicated that the number of polymorphic sites in *A. naeslundii* and monomorphic in *A. oris* was 59 but the number of polymorphic sites in *A. oris* and monomorphic in *A. naeslundii* was 287 (χ^2^=7.011; *P* =0.00081).

**Table 1:** Genetic variation of partial *nanH* sequences of type and reference strains and clinical isolates of *Actinomyces oris* and *Actinomyces naeslundii*

	*A. naeslundii* (*n* =30)	*A. oris* (*n* =74)
G+C content	0.687	0.693
No. of segregating sites, S	147	319
No. of discrete sequences	29	71
d*N*/d*S*	0.2134	0.1466
π	0.0210	0.0296
θ	0.0361	0.0728
Tajima's *D*	−1.6094	−2.0658*
Fu and Li's D*	−1.9615	−3.6456**
Fu and Li's F*	−1.6815	−3.5907**

π and θ, nucleotide diversity.

All analyses were performed using dnasp version 4.5 (http://www.ub.es/dnasp/index.html/).

* *P* <0.05;

** *P* <0.02.

Analysis of the amino acid sequences of the BNRs of the four species is shown in supporting Table S1. There was overall great consistency in the pattern SXDXGXTW within each of the BNRs for all of the species with only single exceptions in the first for BNRs. In the fifth BNR the pattern was SXDXGXSW in all of the isolates except for one *A. johnsonii* isolate which had the sequence SCGNGASW.

The alignment of the amino acid sequences inferred from the partial *nanH* sequences from the four species with that of *S. typhimurium* LT2 demonstrated that the active site identified in the *S. typhimurium* sialidase was recognized in each of the *Actinomyces* sequences. Nine of the 12 amino acids in the *S. typhimurium* active site were identical in all the *Actinomyces* sequences (supporting Fig. S1). The exceptions among the sequences were the met-99 and trp-121 in *S. typhimurium* which were both replaced by serine and leu-175 which was replaced by phenylalanine.

**Fig. 2:**
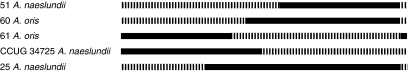
Recombination events found in *nanH* of *Actinomyces oris* strains 60 (EU805671) and 61 (EU805672) and *Actinomyces naeslundii* strains 51 (EU805620), 25 (EU805612) and CUG 34725 (EU805609). Solid line indicates portion of sequence derived from *nanH* of *A. naeslundii* and broken line indicates portion of sequence derived from *nanH* of *A. oris*. Insertion in strain 25 between 365 and 1038, in strain 51 between 629 and 1038, in strain 60 between 1 and 477, in strain 61 between 432 and 1038 and between 1 and 655 in CUG 34725. Breakpoints determined using RDP suite of programs with significant evidence (*P* <0.001) for recombination obtained with ≥5 recombination tests in all cases.

To test for the statistical evidence of recombination in the first instance the Splitstrees method was used and the phi test provided evidence of significant recombination when all strains, except the *A. viscosus* and *A. johnsonii* strains, were included in the analysis (*P* =<10^−20^). Consideration of *A. oris* or *A. naeslundii* strains alone yielded phi values with *P* =3.43 × 10^−6^ and *P* =1.37 × 10^−5^, respectively, indicating statistically significant evidence of recombination in each of the species. To identify the strains with evidence of recombination we analysed the data of these two species together using the seven programs within the RDP suite and found that only *A. naeslundii* strains 25, 51 and CCUG 34725 and *A. oris* strains 60 and 61 gave significant evidence of recombination. The recombination events are summarized in Fig. 2. In the *A. naeslundii* strains 25, 51 and CCUG 34725 and *A. oris* strain 60 the recombination event extended beyond the available partial sequences.

**Fig. 3:**
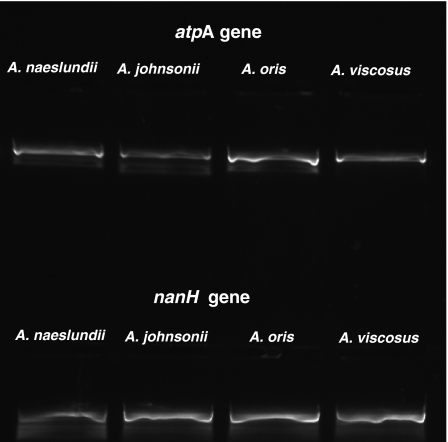
Reverse transcriptase (RT)-PCR assays showing expression of *nanH* and the housekeeping gene *atpA* in the type strains of *Actinomyces oris* (CCUG 34288; EU805602), and *Actinomyces johnsonii* (CCUG 34287; EU805600), *Actinomyces naeslundii* (CCUG 2238; EU805601) and *Actinomyces viscosus* (NCTC 10951; EU805603). Negative controls, omitting the RT in the reaction mix, yielded no amplicons from any strain.

The type strains of each of the four species examined expressed the *nanH* gene when grown on a complex medium containing defibrinated horse blood and 1% (w/v) glucose (Fig. 3) and sequence analysis confirmed that the individual products were derived from the *nanH* genes of the appropriate species.

## Discussion

The complete functions of sialidase in the oral cavity are certainly not fully understood but dietary restriction in macaque monkeys resulted in the increase in levels of sialidase activity in the oral biofilm suggesting a role in bacterial nutrition (Beighton & Smith, 1986). The levels of sialidase-producing bacteria also increase in the absence of host's diet, again supporting the hypothesis that it plays a role in providing nutrient, both C and N, for microbial growth and proliferation (Beighton & Smith, 1986; Lucas *et al.*, 1997; Sheehy *et al.*, 2000). Bacterial sialidases, including those of the oral *Actinomyces*, also expose galactose residues of glycoprotein glycans located on mucosal surfaces or adsorbed to the tooth as part of the enamel pellicle. The exposed galactose residues may then be used as attachment sites by members of the oral biofilm promoting dental plaque accumulation (Costello *et al.*, 1979; Gibbons *et al.*, 1990).

All sialidase enzymes have a highly conserved array of residues, which in the tertiary structure of the enzyme, form the active and binding sites of the molecule (Crennell *et al.*, 1993). In addition to these residues all sialidases also have a number of BNRs structural entities for which a function cannot be definitely ascribed because Asp boxes are found in many protein families (Henningsen *et al.*, 1991; Copley *et al.*, 2001). In determining the sequence diversity of *nanH* in these *Actinomyces* species, we selected a portion of the gene which contained the 12 residues involved in substrate interactions with, and stabilization of, the active site and the BNRs originally described in ‘*A. viscosus*’ strain DSM 43798, now identified as *A. oris*. The gene fragment of 1077 bp represents *c*. 30% of the entire *nanH* sequence in *A. oris*. The determination of the partial sequences of the *nanH* genes from multiple isolates of *A. oris* and *A. naeslundii* has enabled the amino acid conservation within each species and nucleotide sequence variation between the multiple independent strains of *A. oris* and *A. naeslundii* to be determined. The phylogenetic analysis clearly demonstrated that these two species have *nanH* genes with different nucleotide sequences. It was also apparent that the nucleotide sequences of *A. johnsonii* and *A. viscosus* were characteristic and that, despite considerable site-specific conservation of amino acid residues, the amino acid sequences of all four species were distinct and characteristic. The BNRs of the four *Actinomyces* species showed a very high degree of conservation with respect to the amino acid sequence as would be expected (Vimr, 1994). However, in the fifth BNR the threonine residue was replaced by a serine in all the 107 sequences determined; such a structurally synonymous substitution would not be expected to significantly modify the activity of the enzyme. The same variations observed here in the conserved amino acids involved in the active-site region have also been reported in other high G+C organisms (Sakurada *et al.*, 1992; Jost *et al.*, 2001).

The d*N*/d*S* values were <1 for both *A. oris* and *A. naeslundii* indicating that *nanH* in both species is under stabilizing selective pressure; nonsynonymous mutations are not selected. However, for *A. oris* significant negative values Tajima's *D* and Fu and Li's D^*^ and F^*^ were calculated suggesting that, as all nucleotides of any codon are assumed to be equally mutatable, the rate of mutation in *A. oris* must be greater than in *A. naeslundii* but with selection against nonsynonymous mutations. The proposed higher rate of mutation in the *nanH* of *A. oris* was supported by the observation that the frequency of polymorphic sites in *A. oris* which were monomorphic in *A. naeslundii* was significantly greater than the frequency of polymorphic sites in *A. naeslundii* which were monomorphic in *A. oris*. Consideration of our previously published sequence data on six housekeeping genes (Henssge *et al.*, in press) indicated that the frequency of polymorphic sites in *A. oris* which were monomorphic in *A.naeslundii* was also significantly (*P* <0.05) greater in *gltA, metG, pgi* and *rpoB*, while in *gyrA* and *atpA* no significant difference was found. The reasons for the apparent greater frequency of mutations in *A. oris* is not known but may suggest that *A. oris* is subject to greater stress than *A. naeslundii* in the oral biofilm because environmental stress may increase the rate of mutation of organisms in biofilms (Bjedov *et al.*, 2003; Tenaillon *et al.*, 2004). However, given the structural requirements of a functioning sialidase, nonsynonymous mutations may not be beneficial and will, therefore, not be selected. *Actinomyces oris* (as *A. naeslundii* genospecies 2) is numerically more successful in the oral biofilm than *A. naeslundii* (as *A. naeslundii* genospecies 1) (Bowden *et al.*, 1999) which might in part be explained by its higher mutation rate facilitating genome plasticity and an increased ability to respond successfully to environmental stress.

The methods used to interrogate data for the presence of recombinational events provided evidence for significant but limited recombination between the *A. oris* and *A. naeslundii*. This is the first evidence of recombination between these two species although recombination between sialidase genes of other oro-pharyngeal organisms, *Streptococcus oralis* and *Streptococcus pneumoniae*, has been reported (King *et al.*, 2005). We found evidence of recombination in three of 30 *A. naeslundii* strains and in two of 74 *A. oris* isolates suggesting that recombination between these species is either not uncommon or these may be rare events and the recombinants may be more successful than the parent strains. However, the growth or survival benefit acquired by these recombinant strains is not known but because they have persisted and proliferated, at least in the mouths of the individuals from whom they were isolated, this suggests benefit has been accrued. The survival and growth benefits may not be due to recombinational events in *nanH* but to other unknown events, congruent with possession of a recombinant *nanH* gene, which might be responsible for the proliferation of these particular strains.

## Note

*nanH* partial gene sequences have been deposited in GenBank with accession numbers EU805600–EU805702.

## Supporting Information

**Table S1.** Comparison of the amino acid sequence of bacterial neuraminidase repeats [BNRs] and frequency of detection amongst A. naeslundii, A. oris, A. johnsonni and A. viscosus strains.

**Fig. S1.** Alignment of partial sequences of the nanH gene of the type strains of A. naeslundii (accession number EU805601), A. oris (EU805602), A. johnsonii (EU805600) and A. viscosus (EU805603) with the nanH gene sequence of S. typhimurium (M55342).
