# Supplementary material for: Evidence for recombination between a sialidase (nanH) of Actinomyces naeslundii and Actinomyces oris, previously named ‘Actinomyces naeslundii genospecies 1 and 2’
Source: FEMS Microbiol Lett. 2008 Sep 24;288(2):156–62. doi: 10.1111/j.1574-6968.2008.01336.x (PMC2667307; doi:10.1111/j.1574-6968.2008.01336.x)
Supplement: Supplementary file 1 [file fml0288-0156-SD1.doc]

Table S1. Comparison of the amino acid sequence of bacterial neuraminidase repeats [BNRs] and frequency of detection amongst *A. naeslundii*, *A. oris*, *A. johnsonni* and *A. viscosus* strains. The BNRs were identified using the InterProScan Sequence Search tool (<http://www.ebi.ac.uk/Tools/InterProScan/>).

|  | **Species** | **% of isolates** | **BNR sequence** |
| --- | --- | --- | --- |
| BNR1 | *A. naeslundii* | 96.7 | RRSTDGGKTWSA^a^ |
|  | *A. naeslundii* | 3.3 | RRS**A**DGGKTWSA |
|  | *A. oris* | 93.2 | RRSTDGGKTWSA^a^ |
|  | *A. oris* | 1.4 | RR**F**TDGGKTW**L**A |
|  | *A. oris* | 1.4 | RRSTD**N**GKTWSA |
|  | *A. oris* | 1.4 | RRSTDGGKTW**L**A |
|  | *A. oris* | 1.4 | RRSTDGGKT**C**SA |
|  | *A. johnsonii* | 100 | RRSTDGGKTWSA^a^ |
|  | *A. viscosus* | 100 | RRSTDGGKTWSA^a^ |
| BNR2 | *A. naeslundii* | 93.3 | STSTDNGWTWTH^a^ |
|  | *A. naeslundii* | 3.3 | ST**F**TDNGWTWTH |
|  | *A. naeslundii* | 3.3 | S**S**STDNGWTWTH |
|  | *A. oris* | 100 | STSTDNGWTWTH^a^ |
|  | *A. johnsonii* | 100 | S**S**STDNGWTWTH |
|  | *A. viscosus* | 100 | STSTDNGW**S**WTH |
| BNR3 | *A. naeslundii* | 90 | VYSDDHGKTWQA^a^ |
|  | *A. naeslundii* | 3.3 | IYSNDHG**Q**TWQA |
|  | *A. naeslundii* | 3.3 | VYSDDHGK**N**WQ**V** |
|  | *A. naeslundii* | 3.3 | VYSD**N**HGKTWQA |
|  | *A. oris* | 73 | VYSDDHGKTWQA^a^ |
|  | *A. oris* | 20.2 | VYSDDHG**Q**TWQA |
|  | *A. oris* | 1.4 | VYS**N**DHG**Q**TWQA |
|  | *A. oris* | 1.4 | **I**YS**N**DHGKTWQA |
|  | *A. oris* | 1.4 | VYSD**N**HGKTW**R**A |
|  | *A. oris* | 1.4 | VYS**N**DHGKTWQA |
|  | *A. oris* | 1.4 | **I**YSDDHGKTWQA |
|  | *A. oris* | 1.4 | VYSDDHGKTW**R**A |
|  | *A. johnsonii* | 50 | VYSDDHGKTWQA^a^ |
|  | *A. johnsonii* | 50 | VYSDDHG**Q**TWQA |
|  | *A. viscosus* | 100 | VYSDDHGKTWQA^a^ |
| BNR4 | *A. naeslundii* | 90 | A**R**STDGGQTWSE |
|  | *A. naeslundii* | 6.6 | A**Q**STDGGQTWSE |
|  | *A. naeslundii* | 3.3 | **SK**S**N**DGGQTWSE |
|  | *A. oris* | 89.2 | AHSTDGGQTWSE^a^ |
|  | *A. oris* | 8.1 | A**R**STDGGQTWSE |
|  | *A. oris* | 1.3 | **S**HSTDGGQTWSE |
|  | *A. oris* | 1.3 | A**RF**TDGGQTWSE |
|  | *A. johnsonii* | 100 | A**R**STDGGQTWSE |
|  | *A. viscosus* | 100 | A**Q**STDGGQTWSE |
| BNR5 | *A. naeslundii* | 6.6 | SMSCDDGASWTT^a^ |
|  | *A. naeslundii* | 46.6 | SMSCDNGASW**A**T |
|  | *A. naeslundii* | 36.7 | SMSCDDGASW**V**T |
|  | *A. naeslundii* | 3.3 | SMSCDDGA**T**W**V**T |
|  | *A. naeslundii* | 3.3 | SMSCDDGASW**VA** |
|  | *A. naeslundii* | 3.3 | SMSCD**N**GASW**V**T |
|  | *A. oris* | 91.9 | SMSCDDGASWTT^a^ |
|  | *A. oris* | 2.7 | SMSCDDGASW**K**T |
|  | *A. oris* | 1.3 | SMSCDDGASW**V**T |
|  | *A. oris* | 1.3 | SMSCDDG**V**SWTT |
|  | *A. oris* | 1.3 | SMSCDDGASW**M**T |
|  | *A. oris* | 1.3 | SMSCDDG**P**SWTT |
|  | *A. johnsonii* | 50 | SMSCDDGASWTT^a^ |
|  | *A. johnsonii* | 50 | SMSC**GN**GASW**V**T |
|  | *A. viscosus* | 100 | SMSCD**N**GASW**V**T |

^a^ sequence from *A. oris* MG-1 *nanH* (accession number ANA2709).

Amino acids shown in bold are variants.

Fig. S1. Alignment of partial sequences of the *nanH* gene of the type strains of *A. naeslundii* (accession number EU805601), *A. oris* (EU805602), *A. johnsonii* (EU805600) and *A. viscosus* (EU805603) with the *nanH* gene sequence of *S. typhimurium* (M55342). The numbers refer to the amino acid number in the *S. typhimurium* sequence. The BNRs are shown in bold. For the actinomyces type strains the BNRs were identified using the InterProScan Sequence Search tool (<http://www.ebi.ac.uk/Tools/InterProScan/>) while the sequences for *S. typhimurium* were those given in Crennell *et al*. (1993). The underlined residues are those involved in the active site of sialidase as determined by Crennell *et al*. (1993). Number in brackets indicates residue number in *S. typhimurium* protein.

*A. naeslundii* -VGQHPQAEPAPAPDASTELPASMSEAQHLAGNTATDNYRIPAITTAPNGDLLVSYDERP

*A. johnsonii* -VGQHPQAEPAPAPDASTELPASMSQAQHLAANTASDNYRIPAITTAPNGDLLVSYDERP

*A. oris*  -VGDHPQATPAPAPDASTELPASMSQAQHVAPNTATDNYRIPAITTAPNGDLLISYDERP

*A. viscosus* -VVERPKADPAPAPDASTELPASMSDAQHLAENTATDNYRIPAITTAPNGDLLVSYDERP

*S. typhimurium* (1) MTVEKSVVFKAEGEHFTDQKGNTIVGS---GSGGTTKYFRIPAMCTTSKGTIVVFADAR-

*A. naeslundii* KDNGNGGSDAPNPNHIVQ**RRSADGGKTWSA**PTYIHQGTETGKKVGYSDPSYVVDHQTD--

*A. johnsonii* KDNGNKGGDAPNPNHIVQ**RRSTDGGKTWSA**PTYIHQGTETGQKVGYSDPSYVVDNQTG--

*A. oris*  KDNGNGGSDAPNPNHIVQ**RRSTDGGKTWSA**PTYIHQGTETGKKVGYSDPSYVVDHQTG--

*A. viscosus* RDNGNNGGDSPNPNHIVQ**RRSTDGGKTWSA**PSYIHQGVETGRKVGYSDPSYVVDNQTG--

*S. typhimurium* (57) ---HNTASDQSFID-TAAAR**STDGGKTW**NKKIAIYNDRVNSKLSRVMDPTCIVANIQGRE

*A. naeslundii* TIFNFHVKS--YDQGWSGSKAGTDPENRSIIQAEV**STSTDNGWTWTH**-RTITADI-TKDN

*A. johnsonii* TIFNFHVKS--FDQGWSGSRAGTDPEDRNVIQAEV**SSSTDNGWTWTH**-RTITADI-TKDN

*A. oris*  TIFNFHVKS--YDQGWGGSQAGTDPENRGVIQAEV**STSTDNGWTWTH**-RTITADI-TKDK

*A. viscosus* TIFNFHVKS--FDQGWGHSQAGTDPEDRSVIQAEV**STSTDNGWSWTH**-RTITADI-TRDN

*S. typhimurium* (113)TILVMVGKWNNNDKTWGAYRDKAPDTDWDLV---LYK**STDDGVTF**SKVETNIHDIVTKNG

*A. naeslundii* PWTARFAASGQGIQIQHGAHAGRLVQQYTIRTADGTVQAVS**VYSDDHGKTWQA**--GTPTG

*A. johnsonii* PWVSRFAASGQGIQIRQGAHAGRLVQQYTIKTSAEAVQAVS**VYSDDHGQTWQA**--GTPTG

*A. oris*  PWTARFAASGQGIQIQHGPHAGRLVQQYTIRTAGGAVQAVS**VYSDDHGKTWQA**--GTPIG

*A. viscosus* PWTARFAASGQGIQIHQGPHAGRLVQQYTIRTADGVVQAVS**VYSDDHGQTWQA**--GTPTG

*S. typhimurium* (171)TISAMLGGVGSGLQLNDGKLV-FPVQMVRTKNITTVLNTSFIY**STD-GITW**SLPSGYCEG

*A. naeslundii* TGMDENKVVELSDGSLMLNSRASDGSGFRKV**ARSTDGGQTWSE**-PASDKNLPDSVDNAQI

*A. johnsonii* TGMDENKVVELSDGSLMLNSRASDGTGFRKV**ATSTDGGQTWSE**-PVPDKNLPDSVDNAQI

*A. oris*  TGMDENKVVELSDGSLMLNSRASDGSGFRKV**AHSTDGGQTWSE**-PVSDQNLPDSVDNAQI

*A. viscosus* TGMDENKVVELSDGSLMLNSRASDGTGFRKV**ATSTDGGQTWSE**-PVPDKNLPDSVDNAQI

*S. typhimurium* (229)FG-SENNIIEF-NASLVNNIRNS---GLRRSFE**TKDFGKTW**TEFPPMDK----KVDNRN-

*A. naeslundii* IRAFPNAAPSDPRAKVLLLSH-SPNPKPWSRDRGTI**SMSCDDGASWAT**G-------KVFN

*A. johnsonii* IRPFPNAAPSDPRAKVLLLSH-SPNPRPWSRDRGTI**SMSCGNGASWVT**G-------RVFN

*A. oris*  IRAFPNAAPDDPRAKVLLLSH-SPNPKPWSRDRGTI**SMSCDDGASWTT**S-------KVFH

*A. viscosus* IRPFPNAAPSDPRAKVLLLSH-SPNPRPWSRDRGTI**SMSCDNGASWVT**G-------RVFN

*S. typhimurium* (279)-HGVQGSTITIPSGNKLVAAHSSAQNKNNDYTRSDISLYAHNLYSGEVKLIDAFYPKVGN

*A. naeslundii* ENFVGYTT---IAVQSDGSIGLLSENGNYG-

*A. johnsonii* EKFVGYTT---IAVQSDGSIGLLSE-

*A. oris*  EPFVGYTT---IAVQSDGSIGLLSEDA-

*A. viscosus* EKFVGYTT---IAVQSDGSIGLLSEDRNYGGIWYA-

*S. typhimurium* (337)ASGAGYSCLSYRKNVDKETLYVVYEANGSIEFQDLSRHLPVIKSYN (382)
